# Decidualisation of human endometrial stromal cells is associated with increased expression and secretion of prorenin

**DOI:** 10.1186/s12958-015-0127-8

**Published:** 2015-11-25

**Authors:** Eugenie R. Lumbers, Yu Wang, Sarah J. Delforce, Celine Corbisier de Meaultsart, Philip C. Logan, Murray D. Mitchell, Kirsty G. Pringle

**Affiliations:** School of Biomedical Sciences and Pharmacy, University of Newcastle, Newcastle, Australia and Mothers and Babies Research Centre, Hunter Medical Research Institute, Level 3 East, 1 Kookaburra Circuit, New Lambton Heights, NSW 2305 Australia; The Liggins Institute, University of Auckland, Auckland, New Zealand; University of Queensland Centre for Clinical Research, University of QLD, St Lucia, QLD Australia; Present address: Reproductive Sciences, Department of Obstetrics and Gynecology, School of Medicine, University of California San Francisco, San Francisco, USA

**Keywords:** Prorenin, ATP6AP2, Angiotensinogen, Decidualisation, 5-aza-2’deoxycytidine, Angiotensin, VEGF

## Abstract

**Background:**

In pregnancy, the decidualised endometrium expresses high levels of prorenin and other genes of the renin-angiotensin system (RAS) pathway. In this study we aimed to determined if the RAS was present in endometrial stromal cells and if decidualisation upregulated the expression of prorenin, the prorenin receptor ((P)RR) and associated RAS pathways. Immortalised human endometrial stromal cells (HESCs) can be stimulated to decidualise by combined treatment with medroxyprogesterone acetate (MPA), 17β-estradiol (E_2_) and cAMP (MPA-mix) or with 5-aza-2’-deoxycytidine (AZA), a global demethylating agent.

**Methods:**

HESCs were incubated for 10 days with one of the following treatments: vehicle, MPA-mix, a combination of medroxyprogesterone acetate (MPA) and estradiol-17β alone, or AZA. Messenger RNA abundance and protein levels of prorenin (*REN*), the (P)RR (*ATP6AP2*), angiotensinogen (*AGT*), angiotensin converting enzyme (*ACE*), angiotensin II type 1 receptor (*AGTR1*), vascular endothelial growth factor (VEGF), and plasminogen activator inhibitor-1 (PAI-1) were measured by real-time PCR and ELISA’s, respectively. Promyelocytic zinc finger (*PLZF*) and phospho-inositol-3 kinase (*PIK3R1*) mRNA abundances were also measured.

**Results:**

HESCs expressed the prorenin receptor (*ATP6AP2*)*, REN, AGT, ACE* and low levels of *AGTR1*. MPA-mix and AZA stimulated expression of *REN*. Prorenin protein secretion was increased in MPA-mix treated HESCs. E_2_ + MPA had no effect on any RAS genes. MPA-mix treatment was associated with increased VEGF (*VEGFA*) and PAI-1 (*SERPINE1*) mRNA and VEGF protein.

**Conclusions:**

An endometrial prorenin receptor/renin angiotensin system is activated by decidualisation. Since (P)RR is abundant, the increase in prorenin secretion could have stimulated *VEGF A* and *SERPINE1* expression via Ang II, as both *ACE* and *AGTR1* are present, or by Ang II independent pathways. Activation of the RAS in human endometrium with decidualisation, through stimulation of VEGF expression and secretion, could be critical in establishing an adequate blood supply to the developing maternal placental vascular bed.

## Background

The human endometrium/decidua expresses all of the components of the renin-angiotensin system (RAS) [1, 2] including the prorenin receptor [3]. Morphological studies show dense expression of the RAS in the uterine glandular epithelium and in endometrial stromal cells. Stromal cells express renin, angiotensin converting enzyme (ACE) and the angiotensin II type 1 (AT_1_R) and type 2 (AT_2_R) receptors in a cyclical manner, suggesting they are controlled by the sex hormones estrogen and progesterone [1, 2].

Recently, we have shown that in late gestation, the decidua, which is the transformed endometrial lining of the pregnant human uterus, expresses prorenin mRNA (*REN*) and secretes prorenin [4, 5]. Interestingly, levels of expression of prorenin in the decidua are influenced by the sex of the fetus [5].

**Fig. 1:**
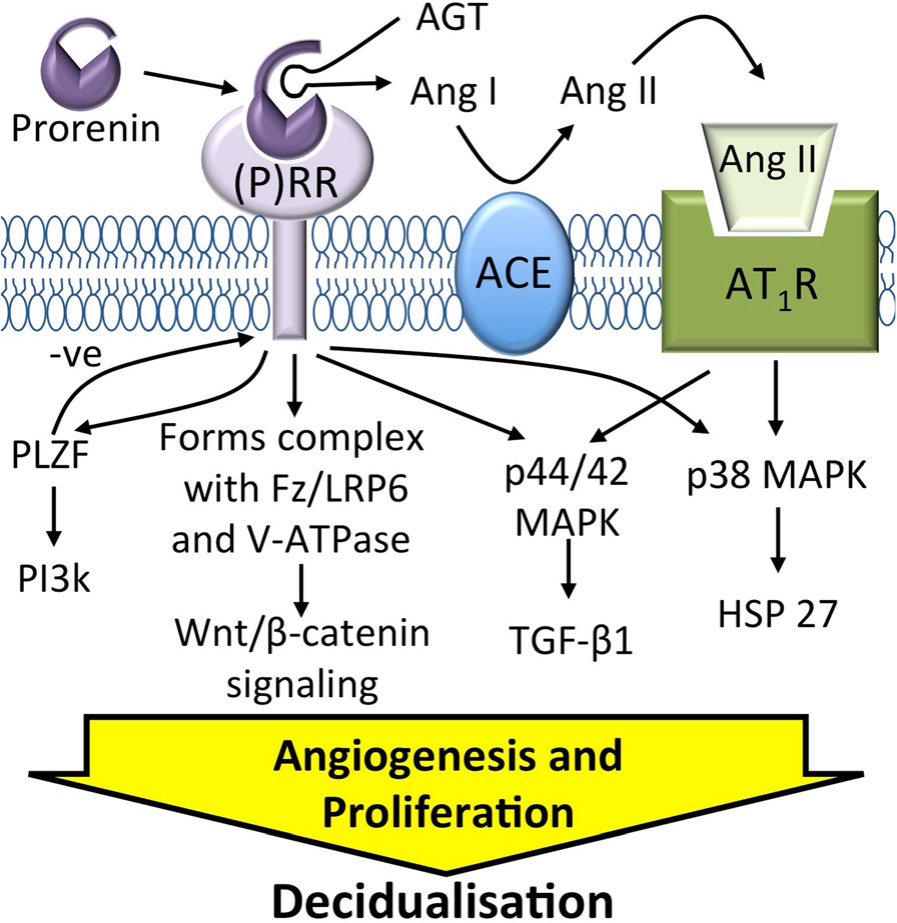
Prorenin/prorenin receptor activated pathways. Prorenin bound to the prorenin receptor ((P)RR) unfolds and can catalyse the formation of Ang I from AGT promoting the formation of downstream Ang peptides such as Ang II. Prorenin bound to the (P)RR activates ERK1/2 signalling, p38/HSP27 signalling, and causes translocation of PLZF to the nucleus which activates p85α-PI3K and down regulates (P)RR expression. The (P)RR can also activate the Wnt/β-catenin cascade

Tissue RASs affect cell growth and proliferation and stimulate angiogenesis. Tissues other than the kidney only secrete prorenin which is inactive and unable to cleave angiotensin I (Ang I) from renin substrate (angiotensinogen, AGT), unless it is activated by proteases, low pH [6] or cold [7]. Alternatively, prorenin can be activated by binding to the prorenin receptor which allows the pro-segment of the enzyme to unfold, exposing the catalytic site [8, 9]. The prorenin receptor is identical to the M8.9 segment (ATP6AP2) of vacuolar H^+^-ATPase (V-ATPase, [9]). Once Ang I is formed, the RAS cascade is initiated, with Ang I conversion to Ang II by angiotensin converting enzyme (ACE, Fig. 1). Ang II is the major biologically active peptide, and via the AT_1_R, is responsible for vasoconstriction, aldosterone secretion, angiogenesis and cell proliferation. Acting via its AT_2_R it has actions that generally oppose the effects of the Ang II-AT_1_R interaction as does the downstream peptide Ang-(1–7) which is formed from Ang II by ACE2.

The prorenin/prorenin receptor interaction not only has the potential to generate Ang peptides in tissues but it has other Ang II-independent actions (Fig. 1). These may be synergistic with Ang II in their effects on angiogenesis, tissue growth and differentiation. These are shown in Fig. 1 and include activation of ERK1/2 and HSP27/p38 signalling pathways, translocation of the transcription factor promyelocytic leukaemia zinc finger protein (PLZF) to the nucleus and subsequent upregulation of the p85α subunit of PI3-kinase, as well as suppression of prorenin receptor gene expression (*ATP6AP2*, [8, 10, 11]). Other actions of the prorenin/prorenin receptor interaction include maintenance of V-ATPase and activation of Wnt signaling [9, 12]. Since decidualisation involves cell growth and proliferation as well as angiogenesis it is very likely that with decidualisation there is upregulation of prorenin/prorenin receptor pathways that promote angiogenesis and cell proliferation.

A human endometrial stromal cell (HESC) line derived from endometrial stromal cells of uterine myomas can be decidualised *in vitro* [13, 14]. Decidualisation is induced by incubation for 9–10 days in medium containing a mixture of medroxyprogesterone acetate (MPA), 17β-estradiol (E_2_), and cyclic AMP (MPA-mix) [14]. HESCs are morphologically transformed from a fibroblast like appearance to large pale glycogen containing cells typical of decidual stromal cells. Furthermore they express prolactin, insulin-like growth factor binding protein-1 (IGFBP-1) and the cytoskeleton protein, desmin when induced to decidualise with MPA-mix [14]. Demethylation of HESCs by 5-aza-2’deoxycytidine (AZA) also induces some of the characteristics of decidualisation [15].

Because there are high levels of expression of RAS genes in late gestation decidua, and there is cyclical expression of RAS proteins in human endometrium, we carried out experiments to find out if the genes of the RAS pathways (Fig. 1) are expressed in this endometrial stromal cell line, whether or not their expression is upregulated when decidualisation is induced, and whether this is mimicked by global demethylation using AZA. We also wanted to find out to what extent the effects of the decidualising ‘cocktail’ on RAS genes depend on E_2_ and MPA independent of any effects of cAMP. Finally we compared the effects of AZA and MPA-mix on the expression of a number of genes and proteins known to stimulated by the prorenin/prorenin receptor interaction and the RAS cascade.

## Methods

### Cell Culture

The hTERT immortalised HESCs (which originated from Dr Charles J Lockwood, Yale University School of Medicine, New Haven, CT, and were a gift from Dr. Peter A.W. Rogers, Monash University) were cultured in Dulbecco’s modified Eagle’s medium and Ham’s F-12 (DMEM/F12) medium (Invitrogen Life Technologies, Auckland, New Zealand), which was supplemented with 10 % heat inactivated fetal bovine serum (Gibco, Invitrogen Life Technologies), 100 mg/ml streptomycin, 100 U/ml penicillin (Invitrogen Life Technologies) and 2 mM glutamax (Invitrogen Life Technologies), and cultured at 37 °C in a humidified 5 % CO_2_ incubator. Cells were detached by trypsinization (1x trypsin-EDTA, Invitrogen Life Technologies).

A mixture of medroxyprogesterone acetate (MPA), 17β-estradiol (E_2_) and dibutyryl cAMP (MPA-mix), in 2 % FBS-supplemented media [16] or 5-aza-2’-deoxycytidine (AZA; Sigma-Aldrich, St Louis, MO, USA) which we know decidualises HESCs, was used [13–15]. In a second series of experiments, an additional treatment with 1 μM MPA plus 10 nM 17β-estradiol but without cAMP was included. The MPA-mix used published concentrations for decidualising endometrial stromal cells [17]. These were 1 μM MPA (Calbiochem, Sigma-Aldrich), 10 nM 17β-estradiol (Sigma-Aldrich) and 0.5 mM cAMP (Sigma-Aldrich) [16]. The AZA concentration was 15 μM, which had been determined by optimization on HESCs by semi-quantitative PCR of prolactin gene expression [15]. A three-way experiment was performed by treating HESCs with AZA or MPA-mix or the control (0.01 % dimethylsulfoxide, DMSO, final concentration).

HESCs were seeded in six-well plates so that 60 % confluent cells were treated the following day. The plates were incubated in a humidified incubator at 37 °C, 5 % CO_2_, and the treatments were renewed with a change of media every 2 days, for 10 days. Total RNA was extracted from HESC cells using TRIZol® (Invitrogen Life Technologies) according to the manufacturer’s instructions, with the addition of an extra chloroform step. Total RNA concentrations were determined by NanoDrop analysis (ND-1000 Spectrophotometer, Thermo Scientific, MA). Total RNA of each sample (1 μg) was DNase treated and converted to cDNA for real-time quantitative PCR (qPCR) using SuperScript III and random hexamers (Invitrogen Life Technologies).

In a second series of experiments, the HESCs were treated similarly, but there were 4 treatments (control, E_2_ + MPA alone, AZA and MPA-mix). In these experiments, media was collected after 2 days incubation and at 10 days when cells were recovered and stored at −80 °C for determination of prorenin protein.

### Real–time quantitative reverse transcriptase polymerase chain reaction (qPCR)

qPCR was performed in an Applied Biosystems 7500 Real Time PCR System using SYBR Green for detection. Each reaction contained cDNA reversed transcribed from 10 ng total RNA, SYBR Green PCR master mix (Applied Biosystems, Carlsbad, CA), RAS primers that we have described previously [3, 18, 19]. The expression of RAS genes: angiotensinogen (*AGT*), prorenin (*REN*), (pro)renin receptor (*ATP6AP2*), angiotensin converting enzyme (*ACE1*) and AT_1_R (*AGTR1*) were examined. In addition, *VEGFA* , *PLZF, TGFB1, PIK3R1* and *SERPINE1* mRNA levels were also measured. Primers for these genes are described in Table 1. Messenger RNA relative abundance was calculated as described previously, using the 2^-ΔΔCT^ method, abundance is expressed relative to both β-actin (*ACTB*) mRNA and a calibrator sample (pooled term decidua for experiment 1, and a term placental sample collected at elective Caesarean section for experiment 2). Therefore, all gene expression data is expressed as a fold change relative to its respective control group.

**Table 1:** Primers used in real time PCR

Gene	GenBank Accession #	Primer Sequence (5’ - 3’)	Conc’n (nM)	Annealing temp (°C)
*PLZF*	NM_000789	F:TAGGGTGCACACAGGTGAGA	200	60
		R:GTGCAGATGGTGCACTGGTA		
*PIK3R1*	NM_181523	F:CGGATCTTGCAGAGCAGTTT	600	75
		R: AGGTTGCTGGAGCTCTGTGT		
*VEGF*	M32977	F:CTACCTCCACCATGCCAAGT	400	75
		R: GCAGTAGCTGCGCTGATAGA		
*SERPINE1*	P05121	F:TCTGTGTCACCGTATCTCA	200	75
		R: GCTCCGTCACGCTGGATGTC		
*TGFB1*	P01137	F:GAACTCATTCAGTCACCATAGCAACACTCT	400	70
		R:TCTCTGGGCTTGTTTCCTCACCTTTA		

F, forward; R, reverse

### Measurement of prorenin, VEGF, PAI-1 and TGF-β1 proteins by ELISA

Prorenin levels in culture media collected every 2 days and measured at 2 days and 10 days using the Human Prorenin ELISA kit (Molecular Innovations Inc; Novi, MI) according to the manufacturer’s instructions. Prorenin in each sample was captured by an antibody immobilised onto the surface of each well of the plate. A primary antibody specific for prorenin was then applied and the unbound fraction removed by washings. For subsequent detection by means of colour development, a secondary antibody conjugated to horseradish peroxidase was used, followed by 3,3,5,5 − tetramethylbenzidine (TMB) substrate. After termination of the reaction with 4 M sulphuric acid, optical density was read at 450 nm. In our laboratory 1 ng/mL amniotic fluid prorenin measured using this technique generated 116 ng/h/mL of Ang I at 37 °C from angiotensinogen present in nephrectomized sheep plasma used as the source of angiotensinogen substrate. All samples were assayed on one ELISA plate. Therefore there was no inter-assay variability. Intra–assay coefficient of variation was 7.3 %.

VEGF, PAI-1 and TGF-β1 concentrations in culture media was measured using the Human VEGF, PAI-1 or TGF-β1 Duoset ELISA kits, respectively (all from R&D systems, Minneapolis, MN) according to the manufacturer's instructions. The protein of interest in each sample was captured by an antibody immobilised onto the surface of each well of the plate. For subsequent detection by means of colour development, a secondary antibody conjugated to horseradish peroxidase was used. Color reagent A (Hydrogen peroxide) and B (3,3,5,5 − tetramethylbenzidine (TMB) substrate) were mixed in equal volumes and added to each well. After termination of the reaction with Stop Solution (2 N sulphuric acid), optical density was read at 450 nm. TGF-β1 samples were activated in order to measure total TGF-β1. Each 100 μL sample was incubated with 20 μL 1 N HCL for 10 minutes at room temperature, after which the reaction was stopped with 20 μL of 1.2 N NaOH/0.5 HEPES. The samples were assayed immediately after activation. The intra-plate coefficient of variation was 7.6 %, 3.8 % and 6.9 % for VEGF, PAI-1 and TGF-β1 respectively. Each assay was run on one plate so there was no inter-assay coefficient of variation.

### Data Analysis

Each experiment was carried out three times in triplicate so each experiment had a vehicle control and a treated set. Data from all 3 experimental sets were combined and univariate analysis with treatments and experiment number as fixed factors carried out using SPSS v21. Tukey’s test was applied to correct for multiple corrections. Significance was set at 5 %.

## Results

### Effects of treatments on genes of the prorenin/prorenin receptor angiotensin system

Both series of experiments showed consistent results; measurable levels of *REN, AGT*, *ATP6AP2* and *ACE* mRNA were found in untreated HESC samples. *AGTR1* levels were low, and low to undetectable results were obtained for *ACE2*.

**Fig. 2:**
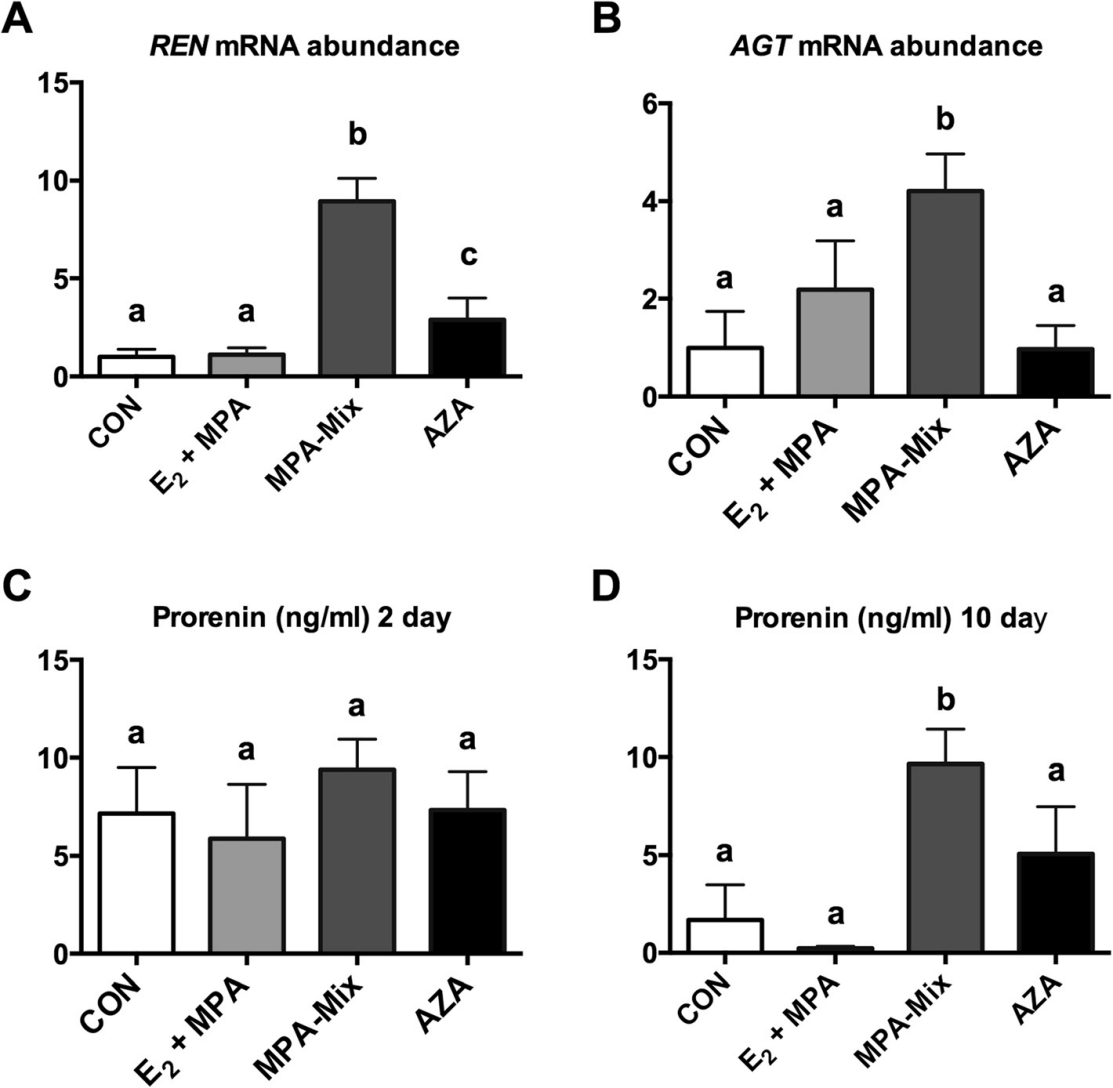
Effects of E_2_ + MPA, MPA-mix and AZA on the renin angiotensin system in HESCs. **a** *REN* mRNA levels were significantly higher in HESCs treated with MPA-mix compared with all other treatments (P < 0.0001). *REN* mRNA expression was also increased in AZA treated HESCs compared with control and E_2_ + MPA treated cells (P < 0.008 and P < 0.014, respectively). **b** *AGT* mRNA levels were significantly increased in MPA-mix treated cells compared with all other treatments (all P < 0.02). **c** Prorenin protein (ng/ml) secreted into media at 2 days. There was no difference between CON and treatments but MPA-mix had significantly more prorenin than E_2_ + MPA alone (P = 0.045). **d** Prorenin protein (ng/ml) secreted into media at 10 days. Prorenin levels were significantly increased in the supernatant at 10 days in MPA-mix treated samples compared with all other treatments (P < 0.022). AZA treatment was associated with increased prorenin levels in supernatant compared with E_2_ + MPA alone (P < 0.015). Different superscripts denote differences between groups. CON, treated with vehicle alone; E_2_ + MPA, treatment with 10 nM 17β-estradiol (E_2_) and 1 μM medroxyprogesterone acetate (MPA); E_2_ + MPA + cAMP, treatment as for E_2_ + MPA plus 0.5 mM cAMP

In both series of experiments treatment with MPA-mix and AZA were associated with upregulation of *REN* relative to vehicle treated controls (P < 0.008, Fig. 2a) and in the second experiment the increase in AGT mRNA abundance was also significant with MPA-mix treatment (Fig. 2b). MPA-mix caused a significantly greater increase in *REN* mRNA than did AZA treatment (P < 0.007, Fig. 2a) in both experiments. AZA treatment was also associated with a 400 % and 170 % increase in *ACE* mRNA relative to vehicle treated controls (P = 0.026 and P < 0.08 respectively). In the second experiment, this increase was significant compared with effects seen in E_2_ + MPA treated cells (P = 0.02). MPA-mix had no effect on *ACE* expression. There were no other effects of either MPA-mix or AZA treatment on the other RAS genes studied in this experiment.

In the second series of experiments, prorenin protein was measured in media of untreated controls at 2 and 10 days incubation. Decidualisation with MPA-mix was associated with significantly greater levels of prorenin at 10 days but not at 2 days (Fig. 2c and d). Comparison of Fig. 2c and d show that prorenin levels in 10 day samples were lower than the concomitant 2 day samples in control and E_2_ + MPA samples (P < 0.001) but were about the same in MPA-mix and AZA treated samples.

### Effects of treatments on genes and proteins known to be responsive to stimulation by the prorenin receptor/prorenin angiotensin system

**Fig. 3:**
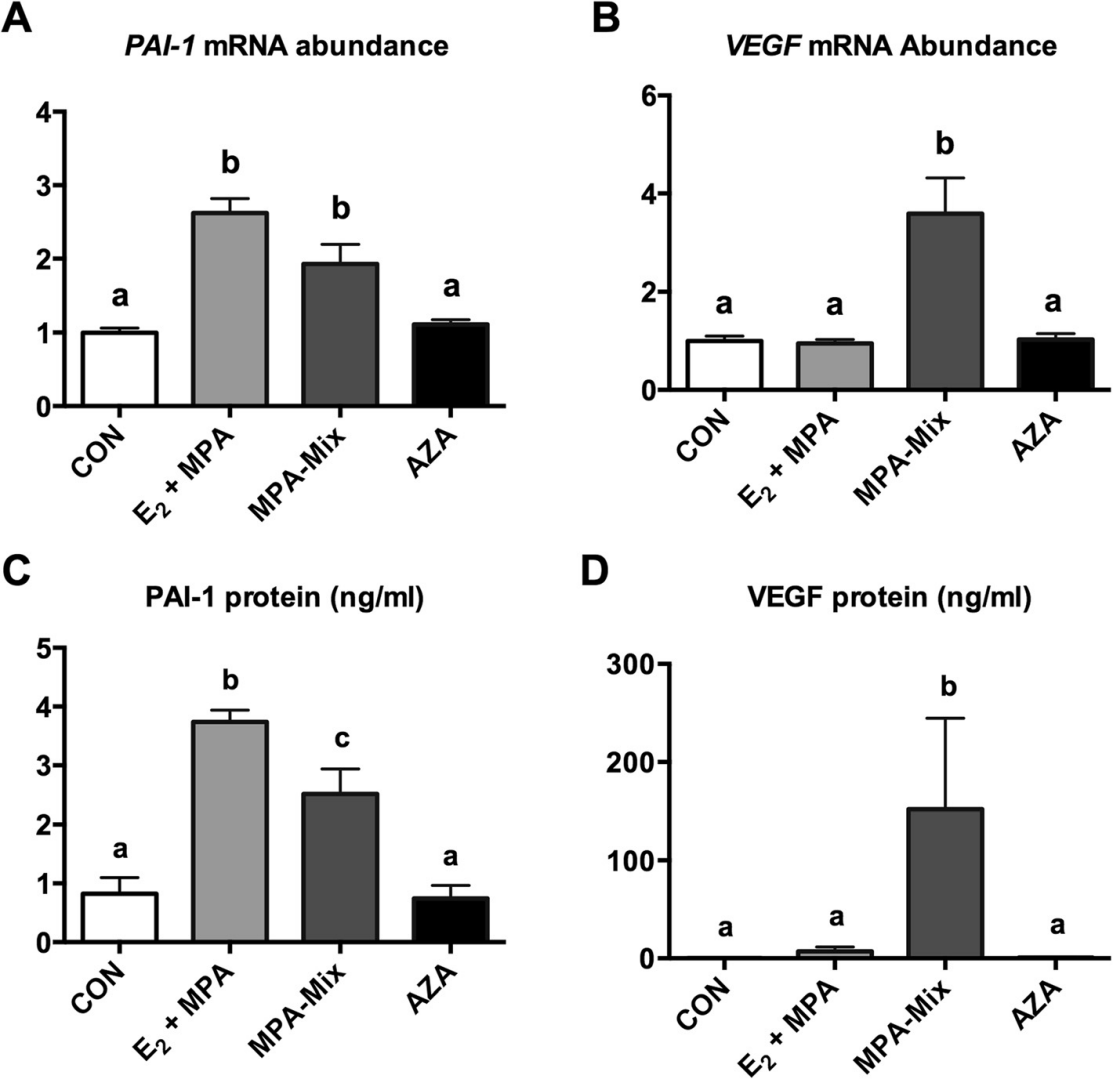
Effects on the expression of genes and proteins known to be responsive to stimulation by the prorenin receptor/prorenin angiotensin system*.* **a** and **c** Relative mRNA abundance of PAI-1 (*SERPINE1*) and its protein. Both E_2_ + MPA alone treatment and MPA-mix treatment stimulated expression of PAI-1(*SERPINE1*) in HESCs (P < 0.001)*.* AZA had no effect. **b** and **d** MPA-mix treatment stimulated the expression of *VEGF A*and its protein compared with all other treatments (all P < 0.001). Different superscripts denote differences between groups. CON, treated with vehicle alone; E_2_ + MPA, treatment with 10 nM 17β-estradiol (E_2_) and 1 μM medroxyprogesterone acetate (MPA); E_2_ + MPA + cAMP, E_2_ + MPA + cAMP, treatment as for E_2_ + MPA plus 0.5 mM cAMP

The effects of the 3 treatments on the expression of *VEGF* and PAI-1 (*SERPINE1*) are shown in Fig. 3. Both E_2_ + MPA alone and MPA-mix stimulated expression of *SERPINE1* in HESCs (P < 0.001*,* Fig. 3a). PAI-1 protein levels were increased in supernatants from both E_2_ + MPA and MPA-mix treated HESCs (P < 0.001) compared with control and AZA treated samples (Fig. 3c). PAI-1 protein levels were higher in E_2_ + MPA treated HESCs compared with MPA-mix treated HESCs (P < 0.006, Fig. 3c). AZA treatment had no effect on *SERPINE1* mRNA abundance nor on PAI-1 protein levels.

MPA-mix stimulated the expression of *VEGF A* (compared with all other treatments P < 0.001, Fig. 3b). VEGF protein levels were also increased in supernatant from HESC treated with MPA-mix compared with all other treatments (P < 0.04, Fig. 3d). AZA treatment had no effect on *VEGF A* expression or VEGF protein levels.

There was no effect of any treatment on the expression of *TGFB1* (data not shown). There was also no effect on total, active and latent TGF-β1 protein levels.

### Effects of treatments on genes known to interact with the prorenin/prorenin receptor system but which have effects independent of formation of Ang peptides

**Fig. 4:**
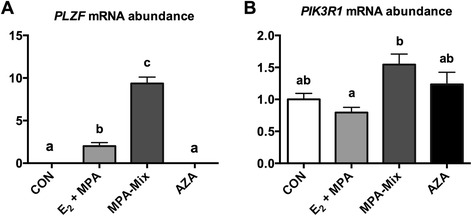
Effect of E_2_ + MPA, MPA-mix (E_2_, MPA + cAMP) and AZA on the expression of genes known to interact with prorenin/prorenin receptor that are independent of Ang II. **a** *PLZF* (Promyelocytic leukaemic zinc finger protein) mRNA was not detected in CON or AZA treated cells but was present in E_2_ + MPA and MPA-mix treated cells (P < 0.001 for both treatments compared with CON and AZA). Expression of *PLZF* in MPA-mix treated cells was much greater than in E_2_ + MPA treated cells (P < 0.001). **b** p85α-PI3kinase (*PIK3R1*) mRNA expression was significantly increased in MPA-mix treated HESCs compared with those treated with E_2_ + MPA alone (P = 0.007) although it was not greater then control (P < 0.07). Different superscripts denote differences between groups. CON, treated with vehicle alone; E_2_ + MPA, treatment with 10 nM 17β-estradiol (E_2_) and 1 μM medroxyprogesterone acetate (MPA); E_2_ + MPA + cAMP, treatment as for E_2_ + MPA plus 0.5 mM cAMP

Two genes were studied; *PLZF* and its downstream target p85α-PI3kinase. *PLZF* mRNA were not detected in control or AZA treated cells but were expressed in both E_2_ + MPA and MPA-mix treated HESCs, although the effect of E_2_ + MPA treatment was significantly less than that seen with MPA-mix (Fig. 4a). AZA treatment had no effect. p85α-PI3kinase (*PIK3R1*) mRNA expression was significantly increased in MPA-mix treated HESCs compared with those treated with E_2_ + MPA alone (P = 0.007) although it was not significantly greater then control (P < 0.07).

## Discussion

The transformation of a stromal endometrial cell to a decidual cell (decidualisation) occurs late in the menstrual cycle under the influence of the hormones estrogen and progesterone. The subsequent accumulation of intracellular cAMP triggers the transformation from a fibroblast phenotype to an inflammatory and finally a secretory phenotype [20]. Previous reports have demonstrated that this can be mimicked *in vitro* as progesterone, either alone or in combination with estradiol, stimulates intracellular cAMP accumulation and decidualisation of human endometrial stromal cells in culture [21, 22]. We have shown that the morphological and molecular changes (increased expression of *FOX01*, *PRL* and *IGFBP-1*) that characterise decidualisation are also recreated in human endometrial stromal cells (HESCs) *in vitro* using MPA-mix [14] and to some extent, by AZA treatment [15]. Overall the stimulatory effects of AZA treatment on the key markers of decidualisation, namely prolactin and IGFBP-1 are much less than effects of MPA mix [14]. Similarly, in this study, the effects of AZA on the renin-angiotensin system and the downstream signalling genes is much less than the effects seen with decidualisation induced by MPA mix.

Despite the presence of renin-angiotensin system proteins in human endometrium, their cyclical expression [2], and the fact that the decidua is the major intrauterine tissue producing prorenin in late gestation [3–5], the effects of decidualisation on expression of the endometrial RAS have not previously been described. In this study we characterized the effects of decidualisation of HESCs on its RAS.

HESCs express *ATP6AP2, REN, AGT, ACE* and low levels of *AGTR1* and *ACE2*. When treated with MPA-mix, expression of *REN* was markedly increased, as was the secretion of prorenin protein (Fig. 2). The effect of MPA-mix on prorenin protein was unlikely due to increased cell proliferation, as we have previously demonstrated that treatment with MPA-mix or AZA significantly inhibits cell proliferation in HESCs *in vitro* [23]. Expression of *AGT* was also increased in the second experiment (in the first experiment the increase in *AGT* did not reach significance). Global demethylation with 5-aza-2’deoxycytidine, which induces decidualisation [15], was also associated with upregulation of *REN* but to a lesser extent than that seen with MPA-mix (Fig. 2). Treatment with AZA had no effect on *AGT* in either experiment but did cause upregulation of *ACE* expression in both experiments. Interestingly, the amount of prorenin in the supernatant was less in control and E_2_ + MPA samples at 10 days compared with 2 days (Fig. 2c and d). This is most likely due to a residual stimulatory effect of fetal bovine serum on prorenin secretion as the cells are cultured in media containing 10 % FBS prior to the experiment and in 2 % FBS during the experiment itself. As the media were changed every 2 days the effect of the experimental treatments, as opposed to FBS can be seen more effectively at 10 days. E_2_ + MPA treatment without cAMP had no effect on *REN*, *AGT* or *ACE* mRNA abundance nor on prorenin protein levels.

In previous studies we have shown that within the intrauterine tissues prorenin expression is highest in the decidua in late gestation [3], that decidual explants grown *ex vivo* for 48 h secrete prorenin, and that both decidual expression of *REN* and prorenin protein levels are highest if the fetus is female [4, 5]. The present studies show that decidual prorenin secretion, which is constitutive, is switched on when stromal cells are decidualised. Thus stromal cells express the renin gene and secrete prorenin, independent of the presence of glandular cells which also contain renin [2] as do placental villous cells which are also responsive to cAMP in terms of *REN* expression and prorenin secretion [24, 25].

Increased expression of prorenin and its secretion in response to MPA-mix, i.e. when decidualisation occurs, is not surprising since cAMP plays a pivotal role in inducing the phenotypic changes that result in transformation of the fibroblast like stromal cell to a secretory decidual cell [20]. cAMP also regulates *REN* expression. Maximal cAMP induction of *REN* depends on a cAMP response element (CRE) in the proximal promoter region that binds cAMP response element binding protein (CREB) as well as a factor interacting with a Pit-1 motif in the *REN* gene [26]. The lack of response to E_2_ + MPA alone in HESCs suggests that these two agents alone did not stimulate cAMP production to an extent sufficient to upregulate *REN* expression (Fig. 2).

cAMP, via a CRE in the AGT gene, also stimulates expression and secretion of AGT by adipose tissue [27]. As well, AGT is stimulated by estradiol through a cis-acting DNA element situated between the TATA box and the start of the transcription site [28]. AGT expression in a number of extra-hepatic tissues is sensitive to E_2_ [29]. It is, therefore not surprising that *AGT* was also stimulated by decidualisation of HESCs. The effect of MPA-mix on *AGT* was however not as consistent as its effect on *REN,* in that the increase in *AGT* mRNA did not reach statistical significance in the first set of experiments; it was only evident in the second set.

In a previous study, Logan *et al.* showed that global demethylation of HESCs with 5-aza-2’deoxycytidine caused down regulation of DNA methyltransferases (DNMTs) and an upregulation of those genes that characterize decidualisation e.g. prolactin, IGFBP-1 and FOX01 [15]. Therefore we studied the effects of AZA-induced decidualisation on expression of HESC RAS genes. In both sets of experiments AZA treatment caused significant upregulation of *REN* expression*.* Levels of prorenin protein secreted by AZA treated HESCs were greater at 10 days than those measured in cells treated with E_2_ + MPA alone but they were not different from control levels. The difference in prorenin protein levels between these two treatments might be accounted for by the decline in prorenin secretion by E_2_ + MPA treated cells.

AZA treatment, unlike treatment with MPA-mix, also appeared to affect the expression of *ACE*. In the first set of experiments *ACE* mRNA was greater in AZA treated HESCs than in vehicle treated controls, while in the second series of experiments *ACE* mRNA abundance was no different from vehicle treated controls but was greater than that measured in E_2_ + MPA treated cells. *ACE* is one of the few genes in the RAS pathway (*ATP6AP2*, *ACE* and *AGTR1*) that have an abundance of CpG islands near the promoter region of the gene. Therefore one might have expected that demethylation by AZA would have upregulated expression of *ACE*. The effect was marginal however and AZA treatment had no effect on *ATP6AP2* nor on *AGTR1* expression.

Prorenin is catalytically inactive *in vivo* unless it is unfolded by low pH treatment or cold, bound to its prorenin receptor or its pro-segment is removed by proteases [6–8, 30]. Assembly of V-ATPase depends on the (P)RR; the absence of the (P)RR leads to decreased expression of Vo subunits of V-ATPase and de-acidification of intracellular vesicles [9]. Since prorenin is susceptible to unfolding in the presence of a low pH [6] it is tempting to suggest that the intimate association between the (P)RR and V-ATPase exposes secreted prorenin to a low pH milieu which unfolds the pro-segment perhaps facilitating binding to the receptor or access by AGT to the catalytic site.

Independent of the RAS, binding of prorenin to the (P)RR activates ERK1/2 pathways stimulating TGF-β1 and PAI-1 production in mesangial cells in culture; it also stimulates proliferation of these cells [31].

Prorenin binding to the cardiac prorenin receptor stimulates HSP27/p38 MAPK [10]. When prorenin alone is applied to cardiac myocytes it does not stimulate ERK 1/2, AGT is also required. In the heart, it is Ang II acting via its AT_1_R that stimulates PAI-1 production by cardiac myocytes [10, 32]. Ang II acting via AT_1_R also stimulates angiogenesis possibly because it stabilizes HIF-1a leading to increased expression of VEGF [33].

Finally binding of prorenin to the prorenin receptor causes translocation of the transcription factor, PLZF, to the nucleus where it stimulates expression of p85α-PI3kinase, causing cell proliferation, and down regulates *ATP6AP2* expression (Fig. 1) [11].

Therefore we examined the expression of 3 downstream pathways that could be activated by the prorenin/prorenin receptor angiotensin system. *VEGF A* and *SERPINE1* expression were increased when HESCs were decidualised with MPA-mix but not when they were treated with AZA. *SERPINE1*expression was also increased when cells were treated with E_2_ + MPA alone. Levels of VEGF and PAI-1 protein reflected the effects of treatment on expression of their genes (Fig. 3). Rather surprisingly no treatment affected the expression of *TGFB1* and levels of either latent or total TGF-β1 protein.

*PLZF* was not detected in vehicle treated control cells nor in AZA treated cells but was expressed in cells treated with E_2_ + MPA and further enhanced by treating with MPA-mix. It is not surprising that PLZF was expressed in both E_2_ + MPA and MPA-mix treated cells because progesterone is known to stimulate the expression of this transcription factor [34]. The effects, if any, of high levels of expression of *PLZF* and increased secretion of prorenin on p85α-PI3kinase expression were marginal in that p85α-PI3kinase mRNA levels were only increased in MPA-mix relative to E_2_ + MPA alone, and not significantly increased compared with control values. We have not however examined phosphorylation of p85α-PI3kinase in this study, which could provide further insight into the possible activation of this pathway during decidualisation.

## Conclusion

In conclusion, HESCs express *ATP6AP2*, *REN*, *AGT* and *ACE*. When treated for 10 days with a cocktail containing cAMP, E_2_ and MPA to induce decidualisation, there is upregulation of *REN* and *AGT* expression and prorenin secretion. Global demethylation of HESC with AZA, which mimics decidualisation [15], also resulted in increased expression of *REN*. Levels of expression of other genes that have been shown to be influenced by the activity of the prorenin receptor/prorenin angiotensin system, namely *VEGF A* and *SERPINE1* were also increased but other effects that can also be attributed to Ang II independent pathways, i.e. stimulation of *TGFB1* and *PIK3R1*,were absent or minimal. High levels of expression of *REN* and increased secretion of prorenin protein when HESCs were decidualised suggests that this system plays a role in regulating cell function in decidua and adjacent tissues throughout pregnancy. In particular, the activation of expression of VEGF and its secretion in association with upregulation of the RAS in HESCs suggests that decidualisation of the endometrial stromal RAS could play a critical role in inducing increased vascularity of the decidua so ensuring an adequate blood flow to the placenta.

## Abbreviations

ACE: Angiotensin-converting enzyme
ACE2: Angiotensin-converting enzyme 2
ACTB: β-actin gene
AGT: Angiotensinogen
AT_1_R/*AGTR1*: Angiotensin II type 1 receptor
Ang: Angiotensin
AT_2_R/*AGTR2*: Angiotensin II type 2 receptor
Ang: Angiotensin
AZA: 5-aza-2’deoxycytidine
cAMP: Cyclic AMP
E_2_: 17β-estradiol
FBS: Fetal bovine serum
HESC: Human endometrial stromal cell line
MPA: Medroxy-progesterone acetate
PAI-1/*SERPINE1*: Plasminogen activator inhibitor-1
PIK3R1: Phosphor-inositol-3 kinase
PLZF: Promyelocytic zinc finger protein
(P)RR/*ATP6AP2*: (Pro)renin receptor
RAS: Renin angiotensin system
*REN*: (pro)renin
TGF-β1/*TGFB1*: Transforming growth factor-β1
V-ATPase: Vacuolar H^+^-ATPase
VEGF: Vascular endothelial growth factor
